# Regulatory CD4^+^ T cells: permanent or temporary suppressors of immunity

**DOI:** 10.3389/fimmu.2024.1293892

**Published:** 2024-02-07

**Authors:** Christian LeGuern, James F. Markmann

**Affiliations:** Center for Transplantation Sciences, Massachusetts General Brigham, Harvard Medical School, Boston, MA, United States

**Keywords:** regulatory T cells (T reg), mechanism, transplantation, inflammation, self-tolerance

## Introduction

Since the onset of their discovery (reviewed in (1)), it is of common understanding that CD4^+^ regulatory T lymphocytes, or Tregs, belong to a critical subset of regulatory immune cells preventing autoimmunity. Although various cell types, including CD8^+^ T cells and monocytes/macrophages, have been described as regulatory cells, this article will focus on the principle of immune regulation mediated by the most studied Tregs, the CD4^+^ Tregs, hereafter referred to as Tregs. The discovery of effective regulatory cells came after a long quest by immunologists who previously described all sorts of T suppressive lymphocytes with no specific biomarkers and elusive suppressive functions (2). Two compelling factors made the scientific community realize CD4 Tregs were the first group of well-defined suppressive cells to control anti-self-reactivity. For the first time, T cell suppressive function was associated with a genetic program involving the Foxp3 transcription factor (3). Foxp3^+^ Treg cells, with suppressive abilities, were found in all mammals. In addition, the Treg control over self-reactivity appeared quite convincing when loss-of-function experiments indicated that the absence of Tregs *in vivo* caused severe autoimmune syndromes (4, 5). These findings ultimately answered the long quest for potential actors of immune regulation, and the interpretation of Tregs as active sentinels preventing autoimmunity became widely accepted.

This concept of Tregs as permanent guardians of immune integrity became a “dogma” in the experimental transplantation arena. Found in many publications, the canonical introductory sentence illustrates the current view: “CD4^+^ Tregs is a critical regulatory cell subset maintaining self-reactivity at bay as depletion of Tregs results in autoimmunity and graft rejection”. However, recent experimental evidence and further analyses of Treg-depleting models may challenge this interpretation. The present report further analyzes observations that initially supported the active Treg sentinel concept and provides new insights from published studies, suggesting that peripheral Tregs are not in a permanent state of activation/suppression.

## Tregs suppress anti-self and -allogeneic responses alike

No evidence suggests that primary autoimmune and alloimmune responses would differ. Indeed, both types of responses implicate T cell clones positively selected on self-antigens and potentially cross-reactive to alloantigens. So, the mechanism of Treg suppression of either response should be similar. The main difference between the two response types and their Treg control relates to the time frame and context of antigen presentation. It is commonly viewed that T cell tolerance to self-antigens is acquired in the thymus during ontogenesis and at early ages along progressive steps of antigen exposure.

In contrast, the implantation of an allogeneic transplant instantly confronts the host’s immune system, including the Tregs, with an extensive array of cross-reactive antigens that can potentially activate up to 10% of a fully developed mature T cell repertoire. The density and duration of antigen exposure to the effector and regulatory T cells may also impact the activation status of Tregs and T effector cells (Teff). For example, self-reactive T cells would not be activated by sequestrated antigens and thus would not require Treg suppression (6). An infection will, on the other hand, generate inflammation and potentially uncover self-antigens.

We may postulate that Treg control of self-reactivity is a progressive process developing during the establishment of the immune system upon gradual antigen exposure. In contrast, Treg-mediated tolerance to transplants would require immediate suppression of recipient T cell reactivity against a large panel of cross-reactive alloantigens well-exposed in inflammatory sites.

## The origin of the “Active Guardian Treg” concept

The seminal work of Shimon Sakaguchi’s team (7) described a new subset of CD4^+^ T cells expressing the IL-2 receptor α chain (CD25) that when “eliminated” “produces a wide spectrum of organ-specific autoimmune diseases, systemic autoimmunity, and GVHD-like wasting disease in normal mice.” The study also demonstrated that adoptive transfers of CD4^+^ CD25^+^ T cells prevented these autoimmune developments. This study strongly suggested that peripheral self-tolerance was constantly maintained, at least in part, by CD4^+^CD25^+^ T cells, dampening the self-reactivity (8). Likewise, the transplantation community shared this analysis and considered Treg cells a promising therapeutic target for transplant tolerance induction (9).

The implication of Tregs in tolerance to self and allogeneic transplants is undisputed. The initial discovery was confirmed in other murine models and humans. It should, however, be noted that although initially reported as activated CD25^+^ regulatory cells (7), contemporary studies indicated that Tregs had the phenotype of anergic cells (10–14). Furthermore, none of the studies provided evidence of constant Treg suppression. Permanent Treg suppression (Treg always ON) would require Tregs to constantly traffic to tissues harboring active anti-self-responses. Conversely, the Treg OFF hypothesis would infer that, following suppression, Tregs will rest. As answers to the Treg ON/OFF dilemma are crucial to understanding immune suppression and designing future Treg tolerance therapies (15), it is vital to reexamine the arguments supporting either side of the quandary and propose alternatives to the currently accepted view on Treg suppression *in vivo*.

## Lessons from CD4^+^ CD25^+^ depletion experiments

Early experiments characterizing this new CD4 ^+^CD25^+^ T cell population evaluated the impact of Treg depletion on self-immunity using thymectomy of neonates or anti-CD25 antibodies. The initial 1995 report from Sakaguchi’s group adequately referred to Treg’s “elimination” over “depletion” since CD25^+^ lymphocytes from the spleen and lymph nodes were killed ex vivo with antibody and complement before injection of the CD25-depleted CD4^+^ cells. Such treatment led to autoimmunity, a result confirmed by other studies ((16, 17)). More importantly, the pioneer experiments used lymphopenic BALB/c nude mice (nu/nu) injected with nu/+ CD4^+^ CD25^neg^ splenocytes in an environment that favors their homeostatic expansion. In mice, leukocyte depletion with anti-CD25 antibodies had differing results according to the age and immune competence of the animals. CD25 depletion significantly accelerated type 1 diabetes in young NOD mice (18) but had no effect in adult mice (19). Similarly, the effective elimination of CD25^+^ cells in adult mice rarely affected the onset of autoimmune gastritis (17, 20). Thus, CD4^+^ CD25^+^ T cells control the onset of autoimmunity in the context of lymphopenia-induced T cell proliferation; conditions found in immunocompromised, thymectomized, or young animals (< 3 weeks) in which the homeostasis of effector and regulatory T cells is still unbalanced (3, 21, 22).

In all these models, it appears that the transient lymphopenic milieu, more than CD4^+^ CD25^+^ T cell depletion, promotes autoimmunity. In support of this eventuality, thymectomy in neonates induces uncontrolled homeostatic proliferation of Teff cells and autoimmunity (23). Recent thymic emigrants from young mice, but not from adults, are highly proliferative in response to TCR engagement to self-pMHC complexes (24). However, an induced period of peripheral lymphopenia in CD4^+^ CD25^+^ depleted animals did not, on its own, affect disease onset (20), suggesting the involvement of other mechanisms in disease initiation. In conclusion, regardless of the importance of CD4^+^CD25^+^ T cells in autoimmunity onset, none of these models provided evidence of the permanence of Treg suppression.

## Clues provided by Foxp3^+^ Treg depletion experiments

Because Foxp3, the critical biomarker of Tregs, is expressed intracellularly, depleting approaches were devised using Foxp3 gene tagging. The initial model was that of the scurfy (sf) mouse in which a natural frameshift mutation in the Foxp3 gene resulted in the absence of Tregs (4). Scurfy mice develop autoimmune disorders affecting primarily lymphoid organs and skin by two weeks of age. Mice die around four weeks from a lymphoproliferative autoimmune syndrome (25). Remarkably, the sf mutation is not embryonic lethal. Despite their immune dysfunction and short life, sf mice do resist opportunistic infections, suggesting that Tregs are not the sole and permanent suppressors of immune reactivity.

Attempts at Foxp3-specific targeting *in vivo* include the development of the B6.Foxp3^hCD2^ mouse in which Foxp3^+^ Tregs are coexpressing the human CD2 antigen that a huCD2-specific and depleting antibody can trap (26). Unfortunately, the claim of sustained transplantation tolerance established by Tregs could not be validated in this experimental setting. It was not an actual Treg depletion *in vivo* but rather a reconstitution of immunodeficient RAG -/- mice with Teff and Treg cells. In the DEREG model, the Foxp3 promoter region is flanked by a fusion gene for eGFP and the diphtheria toxin receptor (DTR) (27). Treatment of DEREG mice with the toxin eliminated 92-98% of GFP^+^ Tregs but only in neonates, leaving us with the same concern of unbalanced homeostasis promoting Teff cell proliferation.

*In vivo*, Treg depletion models derived from mutant mouse strains expressing the DTR only on Foxp3^+^ Tregs have been widely used (5). Multiple observations have converged to similar conclusions: depletion of Foxp3^+^ Tregs is effective but transient upon a short treatment with the toxin. DT administration in 3-month-old mice leads to irreversible multiple organ damage compatible with massive autoimmune responses (28), consistent with the notion that Tregs are constant guardians for life against autoreactivity. Several points, however, deserve further scrutiny before reaching such conclusions. For example, early Treg cell depletion led to massive expansion of myeloid populations (dendritic cells, macrophages, neutrophils, and natural killer cells), which could be due to the overproduction of cytokines by activated CD4^+^ T cells potentially responsible for the autoimmunity pathology observed (29). More recent studies have confirmed this eventuality in the Foxp3-DTR model, showing that DT treatment induces acute T cell lymphopenia in secondary lymphoid organs and non-lymphoid tissues that precede self-reactive T cell expansion (30).

Contrary to scurfy mice, the infiltration of inflammatory cells affects multiple organs in treated Foxp3 DTR mice, including the lung and the central nervous system, possibly attesting to the involvement of additional mechanisms to Treg suppression. Other DTR models, such as those targeting dendritic cells (CD11c-DTR), have also reported important neutrophilia, which on its own could promote the observed inflammation (31). The fact that the effect of Foxp3-DTR Treg depletion finds alternative explanations outside of Treg suppression makes us concur with the later study author’s assessment: “Results from experiments in such transgenic models, especially in mice allowing conditional ablation of immunosuppressive cells, might be prone to misinterpretation.”.

## Evidence supporting the “Treg Guardians for Life” hypothesis

If Tregs constantly suppress the effector’s arm of autoreactivity, Treg-Teff complexes might be detected in protected tissues. Indeed, this has been reported using a transgenic model of TCR Teff cells and multiplex quantitative imaging (32). The study described Treg-Teff complexes in lymphoid and non-lymphoid organs, showing stat5^+^ activated Tregs in interaction with IL-2 secreting Teff cells. Further studies are, however, needed to confirm this observation because the cell-cell complexes are rare events potentially related to the suppression of outliers or recent thymic emigrants that may have escaped negative selection.

Another study reported the cell-targeted depletion of GFP^+^ Tregs following injection of CD8 clones from the Jedi mouse (transgenic GFP-specific CD8 T cells. (33)). Results showed complete depletion of Foxp3-GFP cells in adult mice that rapidly developed clear indications of immune dysregulation (conjunctivitis, splenomegaly, enlarged lymph nodes, and marked expansion of neutrophils). Again, exacerbated neutrophilia could be responsible for cytokine release and activation/expansion of damaging Teff cells. In addition, the depletion of GFP^+^ Tregs by highly reactive cytotoxic CD8 T cells may likely be associated with inflammation, confounding data interpretation.

## A hypothesis: Tregs guard against self and alloreactivity, but only on request

Ultimate answers to the Treg ON/OFF dilemma have yet to be provided. Deciding clues should come from experimental settings that do not rely on specific Treg depletion but instead on *in situ* Treg inactivation while preserving the integrity of their cellular and molecular environment. The recent work of Lim, S.A., and colleagues (34) provides the first tangible indication in favor of Treg transient suppression. The authors utilize a tumor model in which anti-tumor T cells are unresponsive due partly to active Treg suppression within the tumor environment. They demonstrate that the selective inactivation of Tregs *in vivo*, by inhibiting lipid synthesis and metabolic signaling, unleashed anti-tumor responses without causing autoimmunity.

**Figure 1:**
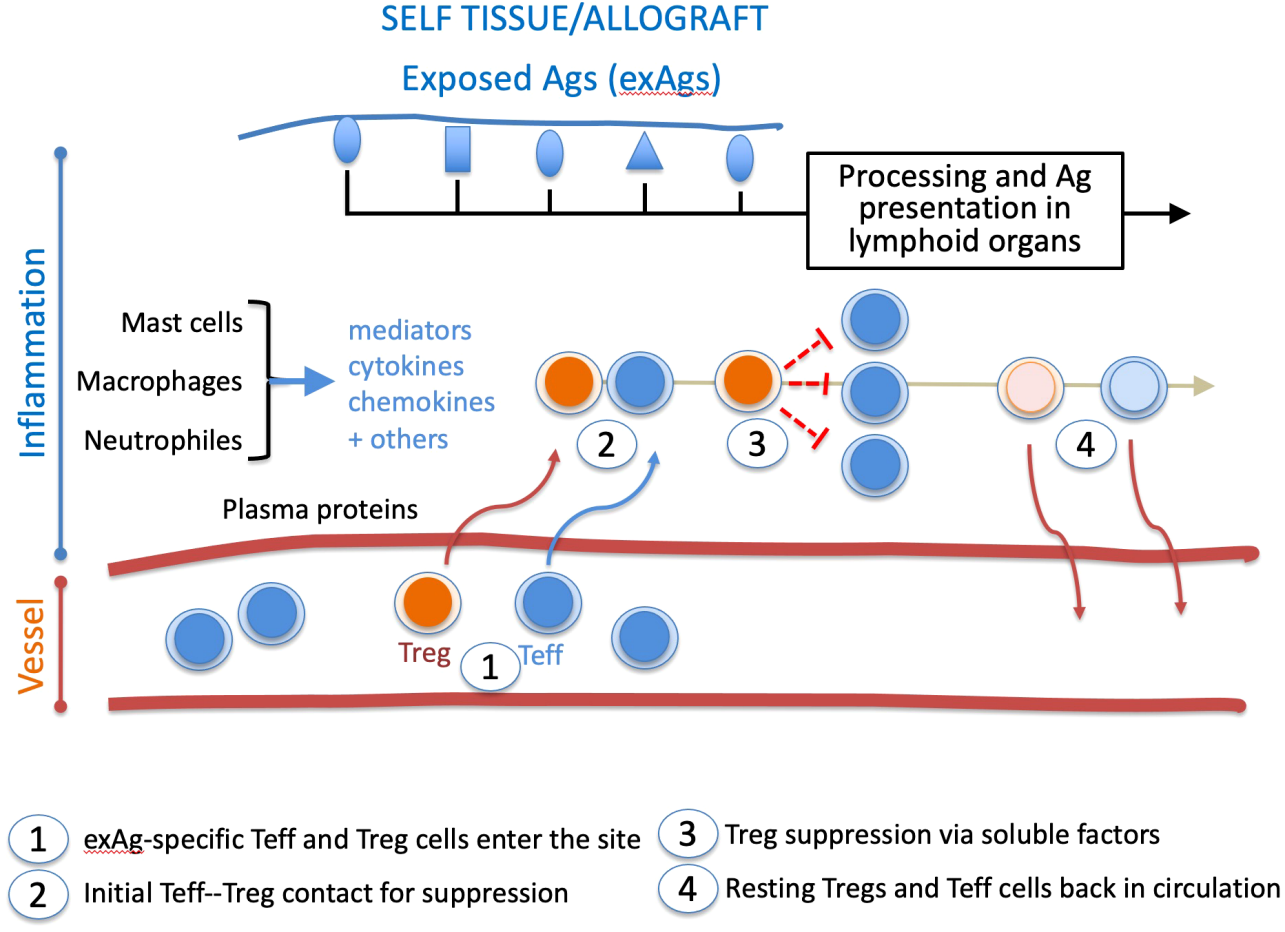
*Chronology of Treg and Teff cell intervention in the inflammatory site*. During inflammation, tissue damage exposes self or allogeneic antigens (exAg). The milieu is conditioned by plasma proteins, mediators, cytokines, and chemokines. It recruits exAg-specific Teff and Treg cells activated in lymphoid organs. In the healing inflammatory site, long-lived Tregs gradually return to a resting state, whereas suppressed Teff cells either die or go to anergy. Both cell types are then released from the healing inflammatory site. This suppressive pathway may apply to other effector cell types like B lymphocytes and antigen-presenting cells.

In the aftermath of this later finding, we should add that Tregs infiltrate graft and draining lymph nodes in places of inflammation (35, 36) to mediate suppression that requires TCR activation (37), initial cell-cell contacts with the target cell (38), and which lessens overtime in inflammatory sites (39). These features suggest that the inflammation sites are the platforms of Treg suppressive mechanisms (**Figure 1**). More precisely, Teff and Treg cells interact in confined environments that prevent systemic Treg suppression while allowing on-demand dampening of activated effector responses. In such situations, constant Treg suppression would not be required, and suppressed Teff cells could survive as anergic cells. This view is supported by findings of self-reactive CD8^+^ T cells with a Treg-induced anergic profile in healthy individuals (40).

This report has commented on and discussed novel interpretations of undisputed data on Tregs’ downregulation of effector pathways. These new insights would let us conclude that the most common justification for permanent Treg suppression (i.e., Treg depletion leads to autoimmunity) does not convincingly support the claim of “always ON Tregs.” Although further studies are required, data acquired so far are compatible with Treg cells acting on demand in local inflammatory sites, i.e., upon request local suppression of only activated effector cells. At steady state (no self- or allo-responses), Treg cells would be inactive. If confirmed, this important mechanism should foster the development of new clinical protocols to improve the timing of Treg activation and their trafficking to inflammatory sites.

## Author contributions

CL: Funding acquisition, Writing – original draft, Writing – review & editing. JM: Funding acquisition, Writing – review & editing.
